# Synthetic Toll Like Receptor-4 (TLR-4) Agonist Peptides as a Novel Class of Adjuvants

**DOI:** 10.1371/journal.pone.0030839

**Published:** 2012-02-20

**Authors:** Arulkumaran Shanmugam, Shilpi Rajoria, Andrea L. George, Abraham Mittelman, Robert Suriano, Raj K. Tiwari

**Affiliations:** Department of Microbiology and Immunology, New York Medical College, Valhalla, New York, United States of America; The Scripps Research Institute, United States of America

## Abstract

**Background:**

Adjuvants serve as catalysts of the innate immune response by initiating a localized site of inflammation that is mitigated by the interactions between antigens and toll like receptor (TLR) proteins. Currently, the majority of vaccines are formulated with aluminum based adjuvants, which are associated with various side effects. In an effort to develop a new class of adjuvants, agonists of TLR proteins, such as bacterial products, would be natural candidates. Lipopolysaccharide (LPS), a major structural component of gram negative bacteria cell walls, induces the systemic inflammation observed in septic shock by interacting with TLR-4. The use of synthetic peptides of LPS or TLR-4 agonists, which mimic the interaction between TLR-4 and LPS, can potentially regulate cellular signal transduction pathways such that a localized inflammatory response is achieved similar to that generated by adjuvants.

**Methodology/Principal Findings:**

We report the identification and activity of several peptides isolated using phage display combinatorial peptide technology, which functionally mimicked LPS. The activity of the LPS-TLR-4 interaction was assessed by NF-κB nuclear translocation analyses in HEK-BLUE™-4 cells, a cell culture model that expresses only TLR-4, and the murine macrophage cell line, RAW264.7. Furthermore, the LPS peptide mimics were capable of inducing inflammatory cytokine secretion from RAW264.7 cells. Lastly, ELISA analysis of serum from vaccinated BALB/c mice revealed that the LPS peptide mimics act as a functional adjuvant.

**Conclusions/Significance:**

Our data demonstrate the identification of synthetic peptides that mimic LPS by interacting with TLR-4. This LPS mimotope-TLR-4 interaction will allow for the development and use of these peptides as a new class of adjuvants, namely TLR-4 agonists.

## Introduction

Lipopolysaccharide (LPS) is the major structural component of gram negative bacteria and is composed of three distinct domains; lipid A, a core oligosaccharide chain, and an O-antigen [1], [2]. Of all three LPS domains discussed, the O-antigen is of most significance as it distinguishes between various gram negative bacterial strains and most importantly, it is recognized by the immune system during infection [2], [3]. It is this recognition and interaction between LPS and the immune system, specifically the innate branch of the immune system, which leads to a potentially life threatening condition known as sepsis. Septic shock remains the number one cause of death in intensive care units and is responsible for 750,000 new cases with 250,000 of these new cases resulting in death within the US [4], [5], [6]. Death by septic shock is attributed to the inflammatory cytokines released by members of the innate immune system, such as antigen presenting cells (APC), which ultimately leads to dysfunction and failure of the body's major organ systems [7], [8], [9].

Inflammatory cytokine secretion occurs upon the initial interaction between LPS and its receptor, toll like receptor 4 (TLR-4), present on APCs such as macrophages and dendritic cells [10], [11]. TLR-4 belongs to a family of transmembrane receptors, originally identified in *Drosophila* [12], which are responsible for recognizing and responding to pathogen associated molecular patterns (PAMPS) such as LPS [2]. Upon activation of TLR-4, a series of signal transduction events occur ultimately leading to nuclear translocation of transcription factor NF-κB, subsequently resulting in transcription of various inflammatory cytokines such as TNF-α, IL-1β, and IL-12p70 [13], [14]. Collectively, these inflammatory cytokines are responsible for the systemic inflammatory response syndrome [7], [9] observed during septic shock. Although responsible for causing systemic inflammation, the inflammatory response associated with LPS may be beneficial if the signal transduction initiated by LPS, which is similar to that observed in adjuvants, can be optimally regulated.

Adjuvants are substances that accelerate and/or enhance an antigen specific immune response as antigens alone are not recognized by the immune system [15]. Thus the sole purpose of an adjuvant is to make an antigen visible to the eyes (macrophages/dendritic cells) of the immune system. Recognition of antigens by APCs essentially initiates the critical cascade of events leading to localized inflammation, which recruits APCs and ultimately leads to initiation of a productive cell and/or antibody mediated immune response [15]. Although various adjuvants exist, not all are approved for human use and the adjuvants which are approved are associated with adverse side effects such as malaise and inflammation [15]. Currently, the majority of human vaccines contain aluminum salts as an adjuvant while pharmaceutical companies are developing oil-based adjuvants to be incorporated into vaccines [15]. Another class of adjuvants which is gaining interest is TLR agonists merely because TLRs are the prime initiators of inflammation [16]. Currently, adjuvants which act as agonists to TLR-2, TLR-5, TLR7/8, and TLR-9 are being studied [17]–[20] and one TLR-4 agonist, monophosphoryl lipid A, is FDA approved [21], [22]. Although promising, many of these agonists are bacterial molecules, which brings to light the possible development and/or use of synthetic peptides as TLR agonists and hence adjuvants.

As mentioned, the potent inflammation induced by LPS activation of APCs via TLR-4 suggests LPS to be an optimal adjuvant only if the inflammation remains localized. One way to exploit the inflammatory properties of LPS such that it can be used as an adjuvant but lack its detrimental side effects is through identification of synthetic LPS peptide mimics using Phage display peptide libraries. To date, no studies have used LPS as a target to identify peptides from Phage display libraries but one study identified peptide mimics of *H. influenzae* lipooligosaccharide (LOS) to be possibly developed into a *H. influenzae* vaccine [23]. The study in the present manuscript was designed to identify LPS peptide mimics, using Phage display libraries that can be developed into a new class of synthetic peptide TLR-4 agonist adjuvants, thus eliminating the use of bacterial proteins/lipids. Various peptides were identified that functionally mimicked LPS by inducing nuclear translocation of NF-κB, which was measured by a colorimetric assay involving the use of a TLR-4 expressing transgenic cell line, HEK-BLUE™-4, as well as Western blot and fluorescence microscopy. In addition, because the inflammation induced by LPS is a consequence of inflammatory cytokine release from activated APCs, we utilized the macrophage cell line RAW264.7, which expresses TLR-4, and observed that various inflammatory cytokines are released upon activation by the LPS mimics. Lastly and most significant, the LPS peptide mimics were capable of functioning as adjuvants in *in vivo* immunization experiments as determined by the increased levels of antibodies in mice that received a vaccine containing the LPS peptide mimic and a prostate cancer specific antigen. Therefore, the peptides identified in this study functionally mimic LPS and have the potential to be developed into a novel TLR-4 agonist adjuvant.

## Results

### LPS peptide mimics stimulate TLR-4 and activate HEK-BLUE™ cells

**Figure 1:**
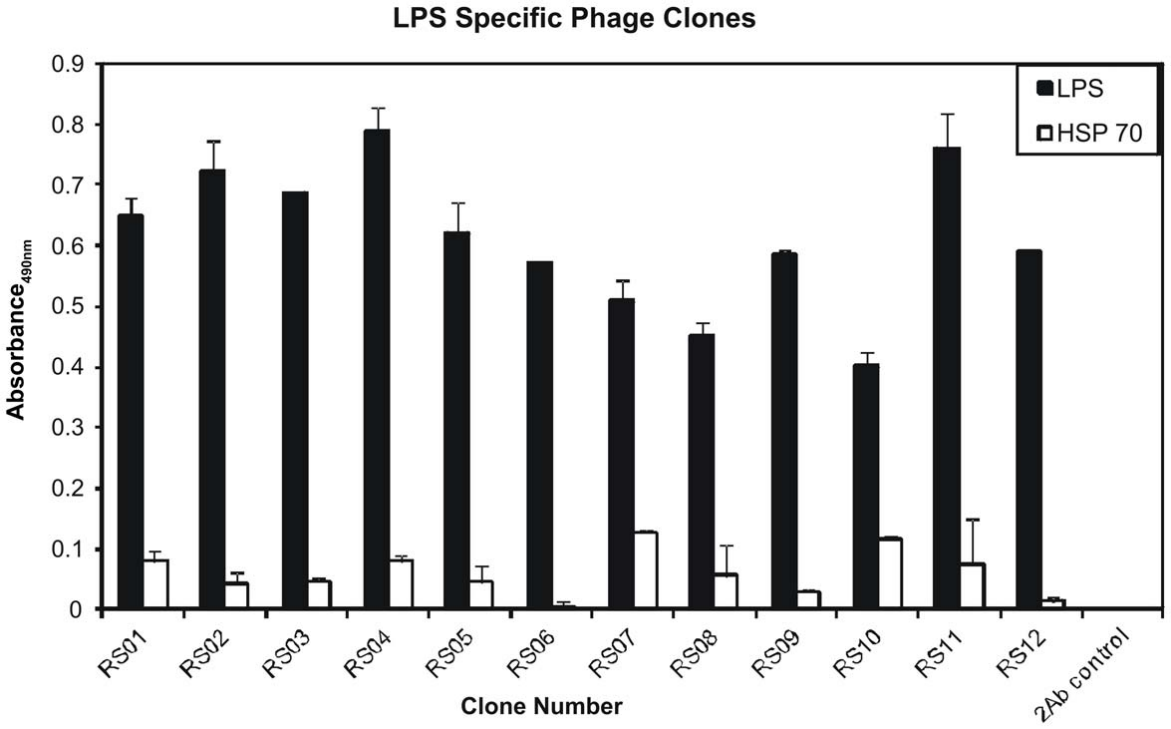
Identification of LPS specific peptide mimotopes. LPS antibody was immobilized at a concentration of 1 µg per well of a 96-well plate. Phages (2×10^11^) expressing 7-mer peptides were initially added to the well of a 96-well plate containing immobilized LPS antibody after which non-specific phages were removed. Twelve random phage clones were selected from three rounds of panning and specificity to LPS antibody (black bars) was confirmed by ELISA using HSP70 antibody (white bars) as a negative control. All 12 (RS01-RS12) clones selected displayed specific reactivity to LPS antibody, as determined using the HRP labeled anti-M13 phage antibody which was detected by the HRP substrate SIGMAFAST™ OPD and absorbance measured at 490 nm. Each experiment was repeated three times with similar results observed and the standard deviation shown above each sample represents three replicates in one experiment.

**Figure 2:**
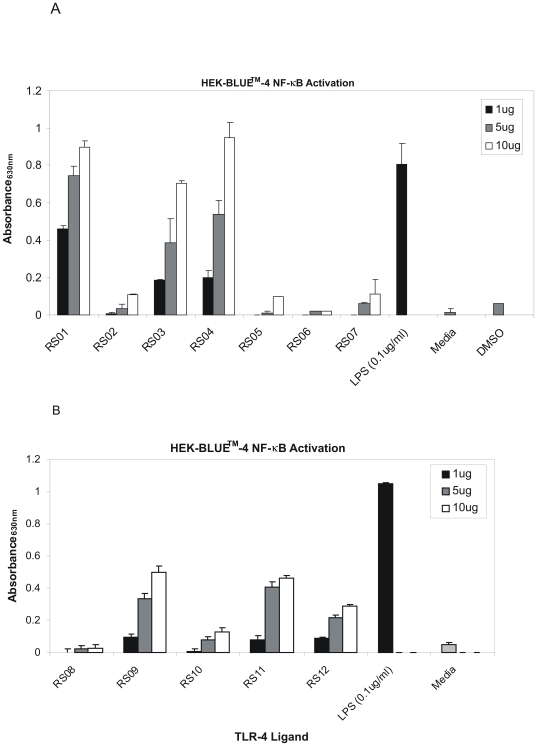
LPS peptide mimics activate NF-κB is HEK-BLUE™-4. LPS peptide mimics were dissolved in endotoxin free water and added to 96-well plates at three concentrations; 1 µg/ml (black bar), 5 µg/ml (gray bar), and 10 µg/ml (white bar). HEK-BLUE™-4 were then added to each well at a concentration of 5×10^4^ cells per well in complete media. The supernatant was then harvested after 24 h and assayed for alkaline phosphatase secretion indicating activation of NF-κB. NF-κB activation for peptides RS01-RS07 is represented in figure 2A and peptides RS08-RS12 in figure 2B. LPS was used as a positive control in assaying all peptides for NF-κB activation. Absorbance was taken at 630 nm. Each experiment was repeated three times with similar results observed and the standard deviation shown above each sample represents three replicates in one experiment.

Phage display technology is a powerful tool used to identify peptides which can be used in many down stream applications such as identification of specific inhibitors or activators of certain target proteins [24], [25]. In this study, Phage display was used for identification of twelve 7-mer peptides (Table 1), which were mimics of LPS as determined by ELISA (Fig. 1). Each clone reacted with the immobilized LPS antibody (black bars) but not with a non-specific antibody such as Hsp70 (white bars), which was used as a negative control (Fig. 1). All twelve peptides were then assayed for their ability to bind to TLR-4 and subsequently activate NF-κB using a transgenic HEK293 cell line known as HEK-BLUE™-4. This cell line is stably transfected to only express TLR-4 on its plasma membrane, with no other TLRs present. Activation of NF-κB is made possible because secreted alkaline phosphatase (SEAP) is under the control of the NF-κB promoter. Therefore, NF-κB activation leads to SEAP secretion, which is detected by an alkaline phosphatase substrate in cell culture media. For all peptides, three concentrations (1 µg/ml (black bars), 5 µg/ml (gray bars), and 10 µg/ml (white bars) were used to activate HEK-BLUE™-4 cells. LPS (0.1 µg/ml) was used as a positive control. Normal media was used as a negative control to assure that it did not contain any substances, such as endogenous alkaline phosphatases, which could be detected by the assay. DMSO was used as a control for peptide RS04 which was not water soluble (Fig. 2A). Of the seven peptides shown in figure 2A, Peptides RS011, RS03, and RS04 were considered as TLR-4 stimulating and hence activators of NF-κB. The activity of peptides RS08-RS12 is shown in figure 2B in which peptides RS09, RS11, and RS12 were considered as positive for TLR-4 stimulation and NF-κB activation.

**Table 1:** LPS Specific Phage Peptide Sequences.

Phage Clone	Peptide Sequence
**RS01**	Gln Glu Ile Asn Ser Ser Tyr
**RS02**	Ser His Pro Arg Leu Ser Ala
**RS03**	Ser Met Pro Asn Pro Met Val
**RS04**	Gly Leu Gln Gln Val Leu Leu
**RS05**	His Glu Leu Ser Val Leu Leu
**RS06**	Tyr Ala Pro Gln Arg Leu Pro
**RS07**	Thr Pro Arg Thr Leu Pro Thr
**RS08**	Ala Pro Val His Ser Ser Ile
**RS09**	Ala Pro Pro His Ala Leu Ser
**RS10**	Thr Phe Ser Asn Arg Phe Ile
**RS11**	Val Val Pro Thr Pro Pro Tyr
**RS12**	Glu Leu Ala Pro Asp Ser Pro

### LPS peptide mimics induce nuclear localization of NF-κB

**Figure 3:**
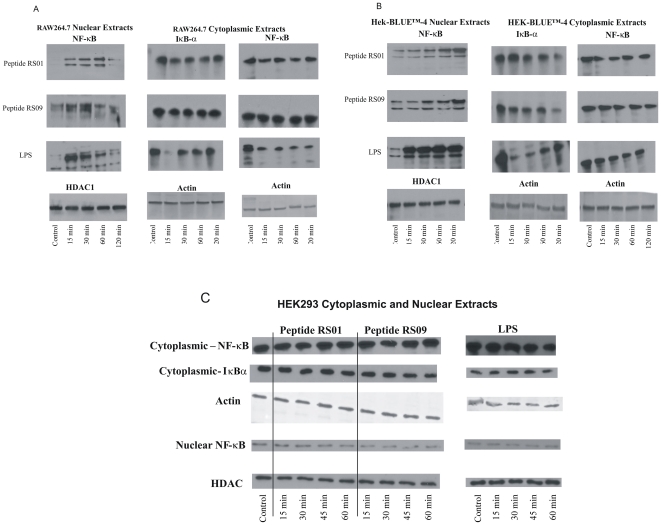
LPS peptide mimics lead to NF-κB nuclear translocation. Nuclear translocation studies of NF-κB were performed by Western blot analysis on both RAW264.7 (Fig. 3A), HEK-BLUE™-4 (Fig. 3B), and HEK293 (Fig. 3C) cells. Cells were stimulated with peptide, either RS01or RS09, at a concentration of 5 µg/ml per 5×10^4^ cells at various time points (15, 30, 60, and 120 min). Nuclear protein fractions were assayed for NF-κB while cytoplasmic proteins fractions were assayed for both NF-κB and IκB-α. HDAC was used as a nuclear protein loading control and actin was used as a cytoplasmic protein loading control. For HEK293 cells, no differences in the levels of cytoplasmic and nuclear NF-kB were observed suggesting that LPS, RS01, and RS09 do not activate the non-TLR-4 expressing HEK293 cells (Fig. 3C).

**Figure 4:**
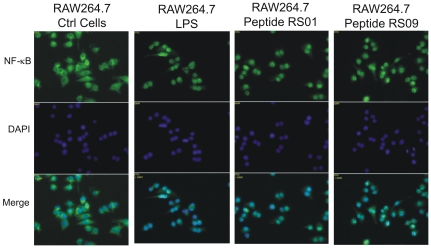
Fluoresence microscopy of NF-κB nuclear translocation. RAW264.7 macrophages were seeded in 8 well chamber slides at a density of 2×10^4^ cells per well and stimulated with 5 µg/ml of either RS01 or RS09. After 30 min incubation, cells were fixed (4% para-formaldehyde), permeabilized (0.2% Triton X-100), blocked (10% goat serum/1% BSA), and incubated with anti-NF-κB followed by DAPI staining of nuclei. The stained cells were visualized using the Axiovert 200 M microscope with a 40× magnification. NF-κB (green) was more diffusely spread throughout the cytoplasm in control cells but appeared more circular in RS01, RS09, and LPS treated cells. Upon merging green NF-κB with DAPI nuclei, nuclear localization of NF-κB was observed by the teal color in treated but not in control cells.

The main event which occurs upon binding of LPS to TLR-4 is nuclear localization of the transcription factor NF-κB, which upon binding to its promoter, results in transcription of inflammatory cytokines; an event at the heart of septic shock [13], [14]. Therefore we wanted to observe if two peptides selected at random, peptide 1 (RS01) and peptide 9 (RS09), were capable of binding to TLR-4 and inducing nuclear localization of NF-κB in the macrophage cell line RAW264.7. We also assayed for nuclear localization in HEK-BLUE™-4 cells side by side with RAW264.7 as a comparison. Both RAW264.7 and HEK-BLUE™-4 cells were incubated with either RS01 or RS09 followed by processing the cells for both cytoplasmic and nuclear protein fractions. LPS was used as a positive control for both cell lines. In RAW264.7, nuclear NF-κB was absent in non-stimulated cells and increased during the indicated times of 15, 30, 60, and 120 min (Fig. 3A). In an inactivated state, NF-κB is sequestered within the cytoplasm by IκB-α, which becomes ubiquinated and degraded upon cellular activation thus releasing NF-κB for nuclear transit. Western blot analysis demonstrated that as nuclear translocation of NF-κB increases over time, so too does the degradation of IκB-α, within the cytoplasm. As expected, concentrations of cytoplasmic NF-κB also decrease, which further attests to the shuttling of NF-κB within the nucleus. Similar results pertaining to increased nuclear localization of NF-κB with simultaneous decrease of cytoplasmic IκB-α and NF-κB were observed with HEK-BLUE™-4 cells (Fig. 3B). For nuclear protein extracts, histone deacetylase 1 (HDAC1) was used as a loading control and for cytoplasmic protein extracts, actin was used as a loading control (Fig. 3A and 3B). Lastly, to demonstrate that RS01 and RS09 indeed bind to TLR-4 leading to NF-κB activation, non-TLR-4 expressing HEK293 cells were used as a negative control. No differences in the levels of NF-κB were observed within the cytoplasm and nucleus suggesting that HEK293 cells treated with either LPS, RS01, or RS09 did not result in nuclear translocation due to the lack of TLR-4 (Fig. 3C). To further demonstrate activation and nuclear translocation of NF-κB in RAW264.7, fluorescence microscopy was performed with peptides RS01 and RS09, with LPS being used as a positive control (Fig. 4). NF-κB in control cells was located within the cytoplasm as observed by the diffuse green staining cells which are better visualized upon the merge with the DAPI stained blue nucleus. When comparing LPS, RS01, and RS09 stimulated RAW264.7 cells, the green staining NF-κB appears more rounded and not diffuse as in the control cells indicating nuclear localization, which was confirmed upon the merge with the DAPI stained blue nucleus resulting in teal colored nuclei (Fig. 4).

### Activated RAW264.7 Macrophages Secrete Inflammatory Cytokines

**Figure 5:**
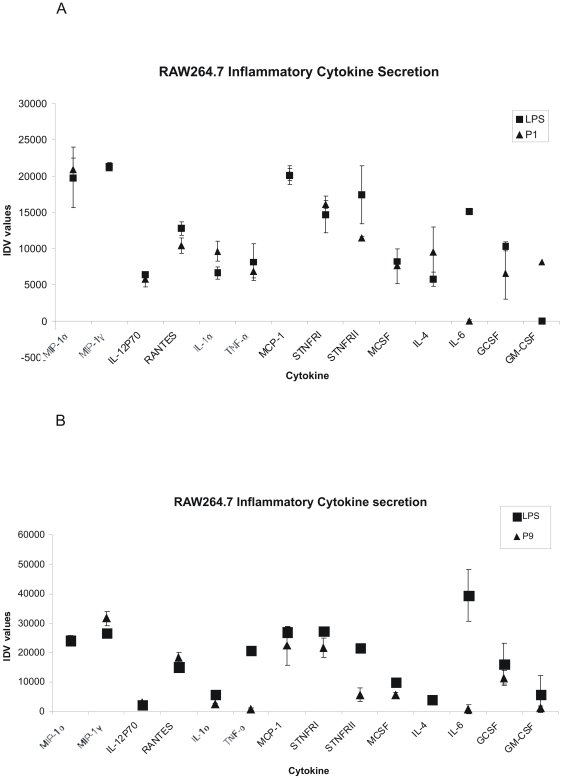
RAW264.7 secretes inflammatory cytokines in response to RS01 and RS09. RAW264.7 cells were seeded at a density of 1×10^6^ cells per well of a six well plate followed by addition of either RS01 (Fig. 5A) or RS09 (Fig. 5B) and incubated for 24 h after which the culture media was collected. LPS was used as a positive control for both RS01 and RS09 and therefore represented on both Figures 5A and 5B. Culture media was then analyzed for specific cytokine using an antibody array kit. Both RS01 (triangle) and LPS (square) were capable of inducing inflammatory cytokines and chemokines from RAW264.7. Each cytokine is represented as an IDV value which was calculated by comparing the density of each spot with respect to the density of the internal positive controls. The antibody array represents a qualitative comparison of each cytokine and is not qualitative. Each experiment was repeated three times with similar results observed and the standard deviation shown above each sample represents three replicates in one experiment.

Inflammation results from binding of nuclear NF-κB to its promoter, thus initiating transcription of various cytokines. Development of the LPS peptide mimics identified in this study as an adjuvant would require them to induce secretion of pro-inflammatory cytokines, which in turn activate the immune system and initiate an antigen specific immune response. Provided that a broad range of cytokines and chemokines are secreted during an inflammatory response, we utilized antibody array membranes to determine which cytokines/chemokines were secreted in response to the LPS peptide mimics RS01 and RS09. Although the values obtained for each cytokine are not quantitative but rather qualitative, they did serve in giving one an idea as to the various inflammatory cytokines secreted. These values were calculated and presented as IDV, which is the density of each spot compared to the density of the internal positive controls present on each membrane. Values for the various cytokines and chemokines detected are presented in Figure 5A for RS01 and Figure 5B for RS09 in which the squares represent cytokines secreted upon LPS stimulation and the triangles represent cytokines secreted upon peptide RS01/RS09 stimulation. Both peptides were capable of inducing secretion of inflammatory cytokines and chemokines from RAW264.7 cells, which was comparable to those secreted from LPS stimulated cells.

### LPS peptide mimics function as adjuvants in vivo

**Figure 6:**
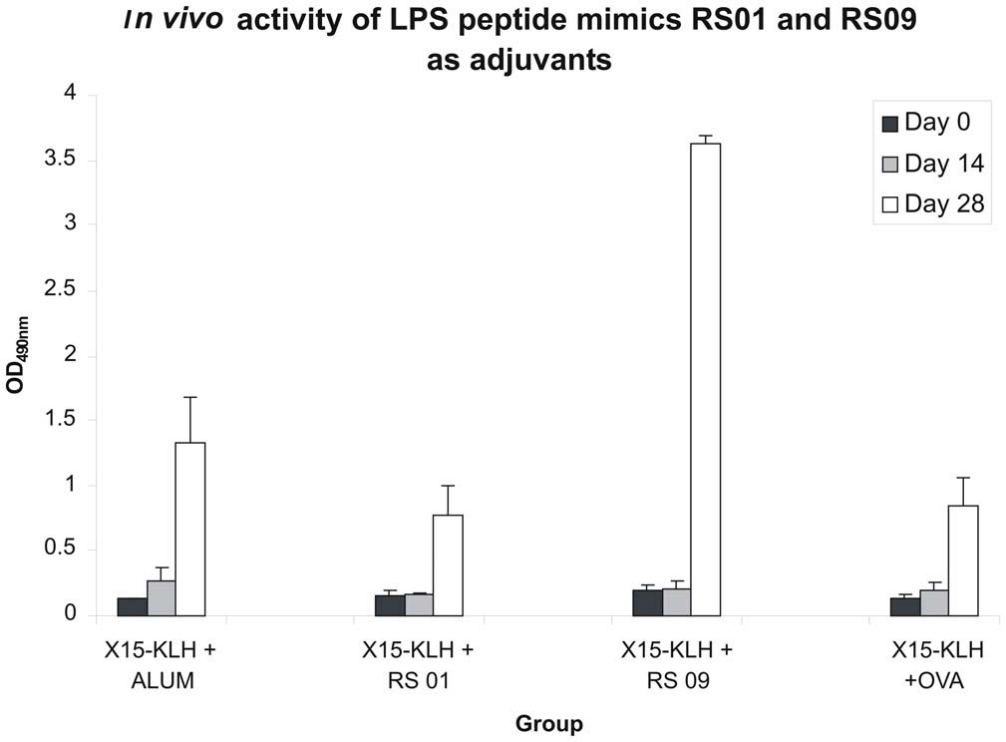
RS09 functions as an adjuvant *in vivo*. Four groups of mice (3 mice/group) were vaccinated with X-15 (100 µg) conjugated to KLH. The mice were bled on days 0, 14, and 28. The serum obtained from each animal was analyzed for presence of X-15 specific antibodies by direct ELISA with immobilized X-15 peptide. The difference between groups was the adjuvant used, which was Alum, RS01 or RS09. OVA peptide (8-mer peptide) was used as a negative peptide control. As shown in figure 6, the highest X-15 specific antibody concentration was observed on Day 28 post vaccination (White bars) for all groups. RS09 was the only peptide capable of enhancing the X-15 specific antibody response on day 28. The standard deviation represents triplicate values for each group of animals.

Weakly or non-immunogenic antigens require an adjuvant that can activate the innate immune system and subsequently initiate an antigen specific immune response. Having observed that the RS01 and RS09 LPS peptide mimics identified in our study were not only capable of inducing NF-κB nuclear translocation but also induced inflammatory cytokine secretion from RAW264.7 macrophages, we next determined if they could function as an adjuvant *in vivo*. The antigen used for our *in vivo* adjuvant study is a 15-mer peptide, known as X-15, which has been observed to possess prostate tumor protective properties *in vivo* when administered with an adjuvant [26]. X-15, conjugated to KLH, was combined with RS01, RS09, or alum and used as an immunogen to vaccinate BALB/c mice. Serum X-15 specific antibody concentrations are shown in Figure 6. Interestingly, despite our observation that both peptides possessed properties typical of an adjuvant *in vitro*, only RS09 was capable of producing a robust X-15 antibody response when compared to Alum and RS01 *in vivo*. This significant increase in X-15 specific antibody concentration with RS09 was observed on day 28 (white bar) post vaccination as shown in Figure 6. RS01 and RS09 were given at a concentration of 25 µg during the initial vaccination on day 0 and during the booster on day 14. Twenty five micrograms was chosen as a starting concentration as this dose, in our experience with using peptides as vaccines, does not induce an antibody response and hence is non-immunogenic. To confirm this observation, two groups of 3 mice were vaccinated with either 25 µg of RS01 or RS09 and no antibodies were produced in these mice (data not shown). This was significant in that RS01 and RS09 were not immunogenic at the concentration used as an adjuvant but were capable, in the case of RS09, of enhancing production of X-15 specific antibodies.

## Discussion

Adjuvants provide a bridge between antigens and APCs of the immune system [27]. In theory, an adjuvant can be considered a catalyst because in its absence, weakly immunogenic or non-immunogenic antigens such as peptides would essentially remain invisible to the immune system. This adjuvant driven immune response is made possible by an inflammatory response, which relies on activation of APCs thus setting the stage for an antigen specific immune response [28]. The inflammatory response is initiated by a cell signal transduction cascade initiated by the TLR proteins, specifically TLR-4, resulting in NF-κB driven transcription of potent inflammatory cytokines and chemokines [29].

Various adjuvants such as QS-21, a saponin derived from the bark of the soap tree, and oil based emulsions are being evaluated in clinical trials for their efficacy. Currently, alum based adjuvants and monophosphoryl lipid A, a bacterial product, remain the only FDA approved adjuvants [15], [21], [22]. Therefore, identification of new adjuvants, such as synthetic TLR-4 agonists, can serve as potential candidates and when considering compounds that can act as agonists of TLR-4, the bacterial cell wall component, LPS, is of prime significance. Classically, LPS is considered a very potent inducer of systemic inflammation, which underlies the condition known as sepsis or septic shock [29], [30]. Despite the negative affects attributed to LPS in septic shock, identification of compounds which can induce inflammation like LPS, but not on a systemic scale, will allow their use as adjuvants.

Identification of compounds which mimic LPS by initiating the essential inflammatory events that are required during an immune response and thus be developed into a potential adjuvant was the goal of this study. Several LPS peptide mimotopes identified in this study were capable of mimicking two essential events that are observed during LPS induced inflammation; namely activation of cells via TLR-4, resulting in nuclear translocation of NF-κB and secretion of inflammatory cytokines. Consistently, all the peptides chosen to assay for their NF-κB activating ability, using the TLR-4 expressing transgenic HEK-BLU™-4, displayed differential activity while other peptides were non-activating. Due to the fact that the HEK-BLU™-4 cell line expresses TLR-4 exclusively, it confirmed that these peptides acted as mimics of LPS. LPS activation of TLR-4, resulting in initiation of a signal transduction cascade culminating in NF-κB activation/nuclear translocation, was also observed to be true for the two peptides assayed for in our study (RS01 and RS09). Both peptides RS01 and RS09 were observed to initiate nuclear translocation of NF-κB in the murine macrophage cell line, RAW264.7 as well as in HEK-BLU™-4. This observation using RAW264.7 was more significant to this study as it is a macrophage cell line and hence interaction with TLR-4 thus ensuing NF-κB activation lend *in vivo* significance and credence for adjuvant activity. Interestingly, for the nuclear translocation experiments, both by Western blotting and fluorescence microscopy, LPS was used as a positive control and displayed similar activity to that of RS01 and RS09 indicating our peptides to be true LPS peptide mimics. It is important to mention that the kinetics of nuclear NF-κB translocation was greater for LPS that both RS01 and RS09 in HEK-BLU™-4 and RAW264.7 cells. This infers that LPS generates a more rapid and robust immune response compared to RS01 and RS09 but this is a favorable observation with respect to developing RS01 and RS09 into adjuvants. Ideally, we want the immuno-stimulatory response of that observed with RS01 and RS09 and not that of LPS, which can potentially lead to an exaggerated immune response as that observed in septic shock syndrome.

One critical event addressed in our study was if our LPS peptide mimics were capable of inducing secretion of cytokines that initiate an inflammatory response. Provided that both RS01 and RS09 were active at the level of NF-κB nuclear translocation, both RS01 and RS09 were assayed for cytokine expression studies. Inflammatory cytokine secretion in RAW264.7 was similar for LPS, RS01, and RS09. The cytokines detected ranged from pro-inflammatory cytokines such as TNF-α, IL-1β, and IL-12p70 to chemokines such as MCSF, GCSF and GM-CSF, which are crucial chemo attractants and activators of macrophages and leukocytes. All of the cytokines detected cooperate in activating the innate immune system, in response to antigen, thus initiating inflammation. Initiation of this inflammatory response is what defines an adjuvant, such as the LPS peptide mimics identified in this study.

Lastly, in order to validate our claim that these LPS peptide mimics can act as adjuvants, *in vivo* experiments were performed in BALB/c mice. Knowing fully well that results observed in *in vitro* experiments do not always correlate or translate into *in vivo* experiments, both peptides (RS01 and RS09) were individually used as an adjuvant in the animal studies and the immunogen used was a prostate cancer specific antigen, X-15. Interestingly, only one peptide, RS09, was capable of increasing X-15 specific antibodies *in vivo*. The fact that RS01 did not react as RS09 *in vivo* needs further examination but can be contributed to various factors. First, it may very well be that the concentration of RS01 used as an adjuvant may not be optimal to activate APCs *in vivo*, thus requiring further experiments with varying RS01 concentrations. Secondly, the RS01 peptide may need to be administered as an emulsion such that it remains localized when administered with an immunogen. In our experiments, both RS01 and RS09 were not formulated as an emulsion with the X-15-KLH immunogen but added in a PBS suspension. Nevertheless, our observation that RS09 increased antibody production in a vaccine setting suggests that this LPS peptide mimic can function as an adjuvant and thus serve as a novel candidate to be considered as a new class of TLR-4 agonist adjuvants.

## Materials and Methods

### Cell Culture

RAW264.7 and HEK293 cells were purchased from ATCC (ATCC, Manassas, VA) and were cultured in DMEM supplemented with 10% fetal bovine serum (FBS) (Atlanta Biologicals, Lawrenceville, GA), penicillin 10,000 IU/ml, streptomycin 10,000 µg/ml (Mediatech, Herndon, VA), Plasmocin™ antibiotic 2.5 mg/ml (Invivogen, San Diego, CA), and 2 mM L-glutamine (Mediatech). HEK-BLUE™-4 cells were purchased from Invivogen and were cultured in DMEM supplemented with 10% fetal bovine serum (FBS) (Atlanta Biologicals), penicillin 10,000 IU/ml, streptomycin 10,000 µg/ml (Mediatech), Normocin™ 100 µg/ml (Invivogen), 1× HEK-BLUE™ selection antibiotics mixture (2 ml/500 ml complete media) (Invivogen), and 2 mM L-glutamine (Mediatech).

### Phage Display

Identification of the LPS peptide mimics was performed by using a 7-mer Phage display peptide library referred to as Ph.D.-7 (New England BioLabs, Ipswich, MA). Panning was performed according to manufactures specifications using a LPS specific antibody as the target. The LPS antibody (Abcam, Cambridge, MA, CAT# ab35654) used for the panning was a monoclonal antibody, which was produced by vaccinating mice with whole *Escherichia coli* O111B4J5 cells. Although it is not known by the manufacturer as to which structural component of LPS is recognized by the antibody, it is specific to LPS and has been used in various publications [31], [32]. Briefly, 1 µg of the LPS antibody was absorbed to one well of a 96 well plate in a total volume of 100 µl of PBS overnight at 4°C. Phages (2×10^11^) were added to the well and allowed to adhere at room temperature after which non-binding phages were washed away and bound phages eluted. Eluted phages were subsequently amplified and used for further panning so as to enrich for LPS antibody specific phages. The panning procedure described was repeated three times. Lastly, twelve random phage clones were selected from three rounds of panning and specificity to LPS antibody (black bars) was confirmed by ELISA using HSP70 antibody (ENZO life sciences, Farmingdale, NY) (white bars) as a negative control. The species for both the anti-LPS and anti-HSP70 antibodies used were mouse. Bound phages were detected by using a HRP labeled anti-M13 phage antibody (Abcam) followed by adding the HRP substrate SIGMAFAST™ OPD (Sigma, St. Louis, MO) and absorbance measured at 490 nm using a microplate reader (Bio-Rad, Hercules, CA). The panning, titer determination, and ELISA experiments were performed according to the manufacture's protocol. Once their reactivity was determined, the DNA encoding the peptide sequence from each positively reacting phage clone was purified using QIAprep Spin M13 Kit (Qiagen, Valencia, CA) and were sequenced by Genewiz (Genewiz, South Plainfield, NJ) using the primers supplied in the kit (New England BioLabs). The LPS peptide mimics sequenced were synthesized by Genemed Synthesis and their purity was greater than 95% (Genemed Synthesis, San Antonio, TX).

### NF-κB Activation Assay

The LPS peptide mimics synthesized were dissolved in endotoxin free water (HyClone) at a concentration of 1 µg/ul with the exception of peptide 4 which was dissolved in a solution of 1% DMSO made in endotoxin free water at a 1 µg/ul concentration. All of the peptide stock solutions and stored at −80°C until they were used. Each of the 12 peptides (RS01-RS12) was added to 96-well plates in triplicates in a total volume of 20 µl. The total volume required in each well was made up with endotoxin free water (HyClone). HEK-BLUE™-4 cells (Invivogen, San Diego, CA), 5×10^4^ cells, were added per well and incubated for approximately 20 h after which the supernatant from each well was collected and placed into a new 96-well plate. One hundred eighty micro liters of HEK-BLUE™ detection media (Invivogen), alkaline phosphatase detection substrate, was added to each well and incubated for 1–3 h at 37°C. After a noticeable color change, the absorbance was taken at 630 nm using the microplate reader (Bio-Rad).

### NF-κB Nuclear Translocation Western Blot

Cytoplasmic and nuclear protein extracts were prepared from HEK-BLUE™-4, HEK293, and RAW264.7 cells (2×10^6^ cells) using NE-PER, nuclear and cytoplasmic extraction reagent kit (PIERCE, Rockford, IL). Manufacturer's protocol was followed to separate cytoplasmic and nuclear fractions. Nuclear and cytoplasmic lysates (10 µg protein) were subjected to 12% SDS–PAGE under reducing conditions (presence of β-mercaptoethanol) as described earlier. Briefly, the proteins were transferred to Immobilon-P membranes (Millipore, Billerica, MA) at 220 mA for 2 h and membranes were blocked with 4% dried milk in TBST [200 mM Tris–HCl, pH 7.4, 150 mM NaCl, and 0.1% Tween-20 added fresh/liter of 1×TBS (TBST)] for at least 2–3 h on a shaker at room temperature. Subsequently, the membranes were incubated overnight at 4°C with either NF-κB p65 (Cell Signaling Technology, Danvers, MA), IκB-α (Cell Signaling Technology), Actin (Santa Cruz Biotechnology, Santa Cruz, CA), or HDAC1 (Santa Cruz Biotechnology) antibodies in TBST on a shaker. Membranes were then washed three times with TBST and then incubated with the respective secondary antibody for 2 hrs at room temperature on a shaker. After four washes with TBST and one wash with TBS, membranes were developed by ECL (Pierce) and detected on HyBlot CL™ autoradiography film (Denville Scientific, Inc, Metuchen, NJ).

### NF-κB Nuclear Translocation Immuno Fluoresence

HEK-BLUE™-4 and RAW264.7 cells were seeded in 8-chamber slides (BD Biosciences, Bedford, MA) at a density of 2×10^4^ cells per well. Cells were allowed to adhere at 37°C over night after which the cells were stimulated with LPS peptide mimics at various time points and subsequently processed for immuno fluoresence. The cells were fixed with a 4% paraformaldehyde/PBS solution, followed by permeabilization with 0.2% triton X-100, and lastly the cells were blocked using a solution of 0.1% triton X-100, 1% BSA, and 10% goat serum. The cells were then incubated with the NF-κB p65 antibody (1∶50) (Cell Signaling) overnight at 4°C followed by incubation for 45 min at room temperature with Alexa Fluor® 488 labeled goat anti-rabbit (1∶250) (Invitrogen, Carlsbad, CA). The slides were then mounted with Vectashield® containing DAPI (Vector Laboratories, Burlingame, CA). Cells were visualized using the Axiovert 200 M microscope (Zeiss, Thornwood, NY)

### Inflammatory Cytokine Detection

RAW264.7 cells were seeded at a density of 1×10^6^ cells per well of a six well plate and allowed to adhere overnight at 37°C. Cells were then stimulated with LPS peptide mimics for 24 h at 37°C after which the cell culture supernatant was collected and assayed for various secreted inflammatory cytokines by using the mouse inflammation antibody array-1 kit (RayBio®, Norcross, GA). Briefly, the membranes provided were blocked with 1× assay diluent followed by incubation with the cell culture supernatant overnight at 4°C. The blots were the incubated with biotin labeled anti-cytokines followed by incubation with avidn-HRP (1∶250). The blots were then developed using ECL (Pierce) and detected on HyBlot CL™ autoradiography film (Denville Scientific). Detailed instructions for detection of inflammatory cytokines were provided by the manufacturer.

### Animal studies to examine adjuvanticity

Eight week old male BALB/c mice (Charles River Laboratories, Wilmington, MA) were divided into 4 groups (3 mice per group). The antigen used in these studies is a 15-mer peptide, which has been observed in our lab to possess anti-prostate cancer activity in rats and produced a robust antibody response *in vivo* when administered with an adjuvant [24]. The X-15 peptide was conjugated to Keyhole Limpet Hemocyanin (KLH) (Pierce) and injected subcutaneously. All the mice were vaccinated with 100 µg of X-15, conjugated to KLH, on day 0 and received a booster of 50 µg of X-15 conjugated to KLH, on day 14. The difference between groups was with the type of adjuvant used, which was Alum, RS01, or RS09. Alum was added at a 1∶1 (v∶v) ratio to X-15-KLH while both RS01 and RS09 were added at a concentration of 25 µg. OVA peptide (8-mer peptide) served as a negative non-adjuvant peptide control and also used at a concentration of 25 µg. Blood was collected on days 0, 14, and 28. The serum collected was used to determine the presence of X-15 specific antibodies via ELISA.

### X-15 antibody ELISA

X-15 peptide specific antibody was determined by direct ELISA. X-15 peptide was re-suspended in a solution of 0.25% glutaraldehyde-PBS and 5 µg peptide/100 µl was added to each well of a 96well micro titer plate and incubated overnight at 4°C. The plates were washed with PBST (phosphate-buffered saline with 0.05% Tween-20) three times and then wells blocked with 5% milk-PBS for 2 h at room temperature. The plates were washed with PBST once. Then, 100 µl of mice sera was added at the given dilution (1∶200) and incubated at room temperature for 2 h. The plates were then washed with PBST four times and 100 µl of horse radish peroxidase (HRP) labeled anti-mouse IgG (1∶5000 in 2% milk) was added to each well for 1 H at room temperature. The plates were washed five times with PBST and developed by adding 100 µl of the HRP substrate, OPD–hydrogen peroxide (Sigma). The reaction was stopped by adding 50 µl of 4 N H_2_SO_4_ and the absorbency was taken at 490 nm and referenced at 405 nm.

**Ethics Statement:** The animal protocol used by our laboratory in order to test the adjuvanticity of the LPS peptide mimics was approved by our academic institution's (New York Medical College) Institutional Animal Care and Use Committee (IACUC), protocol approval number: 62-12-0610. The animals were allowed to acclimate one week prior to initiating the experiment and were at no time deprived of food or water. At the time of experimentation, the animals were anesthetized via 3% isoflurane inhalation and monitored daily to make sure they did not have any adverse side effects. When blood was required for analyses, the animals were once again anesthetized with 3% isoflurane and blood was collected via retro-orbital sinus bleed by the veterinary technicians from the Department of Comparative Medicine of New York Medical College. At all times during the experimental period, animals were not subjected to any stress.
